# Intake of protein, calcium and sodium in public child day care centers

**DOI:** 10.1590/0103-0582201432214613

**Published:** 2014-06

**Authors:** Giovana Longo-Silva, Maysa Helena de A. Toloni, Risia Cristina E. de Menezes, Tatiane Leocádio Temteo, Maria Alice A. Oliveira, Leiko Asakura, Emília Chagas Costa, José Augusto de A. C. Taddei

**Affiliations:** 1 Faculdade de Nutrição da Universidade Federal de Alagoas (UFAL), Maceió, AL, Brasil; 2 Escola Paulista de Medicina da Unifesp, São Paulo, SP, Brasil; 3 Universidade Federal de Pernambuco (UFPE), Recife, PE, Brasil

**Keywords:** child, osteoporosis, calcium, dietary, sodium, dietary, child day care centers, school health

## Abstract

**OBJECTIVE:**

To assess calcium, protein and sodium intake, of children that attend public day-care centers and to compare it with the recommended one.

**METHODS:**

Cross-sectional descriptive study in seven public day care centers of São Paulo city, Southeast Brazil, which enrolled 366 children between 12 and 36 months of age. The data collection occurred between September and December 2010. Each day care center was evaluated for three non-consecutive days, totaling 42 days and 210 meals. Dietary intake was assessed by a direct food weighing method. For the nutritional calculation, DietWin^(r)^ Profissional 2.0 was used, and the adequacy was calculated according to the recommendations of the National School Feeding Program for energy, protein, calcium and sodium. The calcium/protein relation was also calculated, as well as calcium density (mg/1,000kcal).

**RESULTS:**

The energy (406.4kcal), protein (18.2g) and calcium (207.6mg) consumption did not reach the recommended values ​​in all the evaluated day care centers. Sodium intake exceeded up to three times the recommendation. The calcium/protein ratio of 11.7mg/g was less than the adequate one (20mg/g).

**CONCLUSIONS:**

There was inadequacy of calcium, protein and sodium dietary intake, in children attending public day-care centers.

## Abstract (es)

**OBJETIVO:**

Evaluar la ingestión de calcio, proteína y sodio y compararla con la recomendada.

**MÉTODOS:**

Estudio transversal descriptivo realizado en nidos de siete guarderías públicas del municipio de São Paulo, que atendían a 366 niños entre 12 y 36 meses, siendo el periodo de recolección de septiembre a diciembre de 2010. Se evaluó cada guardería durante tres días no consecutivos, totalizando 42 días y 210 comidas. El consumo alimentar fue evaluado por método de pesaje directa de alimentos. Para el cálculo nutricional, se utilizó el *software DietWin Profissional *2.0^(r)^ y la adecuación se calculó según las recomendaciones del Programa Nacional de Alimentación Escolar (PNAE) para energía, proteína, calcio y sodio. La relación calcio/proteína y la densidad de calcio (mg/1.000kcal) también fueron computados.

**RESULTADOS:**

Se constató que el consumo de energía (406,4kcal), de proteína (18,2g) y de calcio (207,6mg) no alcanzó los valores recomendados en todas las guarderías estudiadas, siendo que la ingestión de sodio sobrepasó en hasta tres veces la recomendación. La relación calcio/proteína de 11,7mg/g fue inferior a la adecuada (20mg/g).

**CONCLUSIONES:**

Los resultados de este estudio señalan la inadecuación del consumo alimentar de calcio, proteína y sodio, suministrando subsidios para el conocimiento sobre la ingestión de los nutrientes en la población infantil y para el primer paso de toma de conciencia y evaluación del consumo alimentar de niños insertados en el contexto de guarderías públicas.

## Introduction

In the last century, life expectancy has increased considerably in both developed and developing countries, which has favored a notable increase in the incidence of age-related diseases, among them osteoporosis^(^ 1 ^)^. The risk of osteoporotic fractures throughout life reaches 40% among women and 13% among men^(^ 2 ^)^. Maintaining bone health is, therefore, one of the major challenges of modern Medicine.

Once the disease is established, it is very difficult to reverse it. Despite all health care strategies, the prevention actions should be prioritized, emphasizing the importance of bone mineralization in the early stages of life, since the acquisition of bone mass occurs mainly in the childhood, and the realization of this peak depends on multiple factors, being nutrition one of the most important^(^ 3 ^,^ 4 ^)^.

Although many nutrients are involved in maintaining bone health, calcium is especially important during periods of rapid growth such as infancy and adolescence^(^ 5 ^-^ 7 ^)^, with its fundamental contribution in the prevention of various diseases in later life, such as obesity, hypertension, insulin resistance, kidney stones, and colon cancer^(^ 6 ^,^ 7 ^)^. On the other hand, the reduced intake causes the mineral already used in the bone to be relocated to other vital physiological functions, such as maintenance of calcium levels^(^ 8 ^)^.

Despite the importance to achieve adequate calcium intake for public health, numerous studies indicate that calcium intake and the consumption of dairy products, main sources of this mineral^(^ 6 ^)^, are frequently lower to the recommended for children, especially in the context of public daycare centers^(^ 9 ^-^ 11 ^)^. However, to assess calcium intake, one must consider the influence of other nutrients that act on its absorption and utilization. In this sense, its sufficient intake is added to the adequate consumption of sodium and the calcium-protein ratio, being established as convenient in the proportion of <20mg/g^(^ 12 ^,^ 13 ^)^.

In this context, the aim of this study was to assess the intake of protein, calcium, and sodium, and to compare it to the recommended daily intake, considering it important to promote future nutritional improvements in the school environment.

## Method

This study is part of the "Projeto CrechEficiente - ("*Efficient Daycare" Project*) Impact of training educators in public/philanthropic daycare centers on hygiene and dietary practices and on infant health and nutrition, whose objectives were to capacitate, improve, and update the daycare educators regarding health care an nutrition provided to infants, as well as to evaluate the educators' acquisition of knowledge related to the activities performed. The selection process of daycare centers and the adopted criteria are described in another publication^(^ 14 ^)^. Of the eight selected daycare centers, one was excluded because it presented no interest in participating in the survey in the period of data collection.

The present study is a cross-sectional, descriptive study developed in the 14 nurseries in the seven selected nurseries. Data collection took place from September to December 2010 and was conducted by four post-graduate researchers from Universidade Federal de São Paulo (Unifesp). 

To assess dietary intake, we used the direct weighing of food to ensure the precision and accuracy of the results, considering the average intake of the group. This methodology has been used repeatedly in Brazilian nurseries, thus, constituting an instrument for this purpose^(^ 10 ^,^ 15 ^,^ 16 ^)^.

All institutions worked full time, from Monday to Friday, and we evaluated all catered meals (breakfast, morning snack or hydration, lunch, afternoon snack, and dinner), during three non-consecutive days, totaling 42 days of analysis and 210 meals.

The weighing of solid foods was performed with a Plenna^(r)^ portable digital scale with capacity of 5kg. Liquid foods were measured with the aid of a graduated container (plastic cup) every 50mL and with a capacity of 1000mL.

We weighed three portions of food and/or preparation, randomly selected, to obtain the average number served. We collected uneaten food in plastic bags, considering as discarded any food left in the cup or plate. Also, to obtain the weight of the rejected meal, weights were collected individually for each food and/or preparation, served for breakfast, morning snack, afternoon snack, and dinner, when composed solely of soup. However, at lunch and dinner, meals in which food is served on the plate and mixed, the analysis examined the proportional waste for each food and/or preparation.

Obtaining the mean of food and/or preparations served, we multiplied the value by the number of children in each group. The repetitions were added to the total value, obtaining, thus, the total weight of each food and/or preparation. From this value we subtracted the waste of each food served and, finally, divided by the number of children in each group, obtaining then, the per capita amount of each food and/or preparation. To calculate the nutritional value of the meals, we used the DietWin Profissional 2.0^(r)^ software.

We calculated the percentage of recommendation for calcium, protein, and sodium, considering the reference values set for the National School Meal Program - 70% of the daily nutritional needs^(^ 17 ^)^. 

Due to the absence of standard recipes for preparing soups, milk, and juices, the team followed the preparation, previously weighing all ingredients used and the final yield. The nutritional information of each preparation was also calculated in DietWin Profissional 2.0^(r)^ and, afterwards, inserted as a new preparation on the food list. 

To determine the socioeconomic profile of children attending the institutions analyzed, we applied a questionnaire to parents to identify the family income of each child, obtained through information on wages and other sources of income of all members of the family unit. The sum obtained was expressed in Brazilian Reais and converted in units of the minimum wage (MW) in effect during the study period. We described the simple frequencies and percentages of the distribution per age, gender, and economic class of the children. 

All data obtained were double entered, validated and analyzed using the Epi-Info 2000 statistical software, version 3.4.3. The project was approved by the Research Ethics Committee of Unifesp (n. 0442/10).

## Results

The socioeconomic characteristics of the children enrolled in the daycare centers analyzed are included in Table 1, and it was noteworthy that the gender distribution of children attending the seven daycare centers was homogeneous, with 366 children aged between 12 and 36 months. The economic conditions of the families involved indicate that 62.8% present a family income ranging between one and three minimum wages. 

**Table 1:**
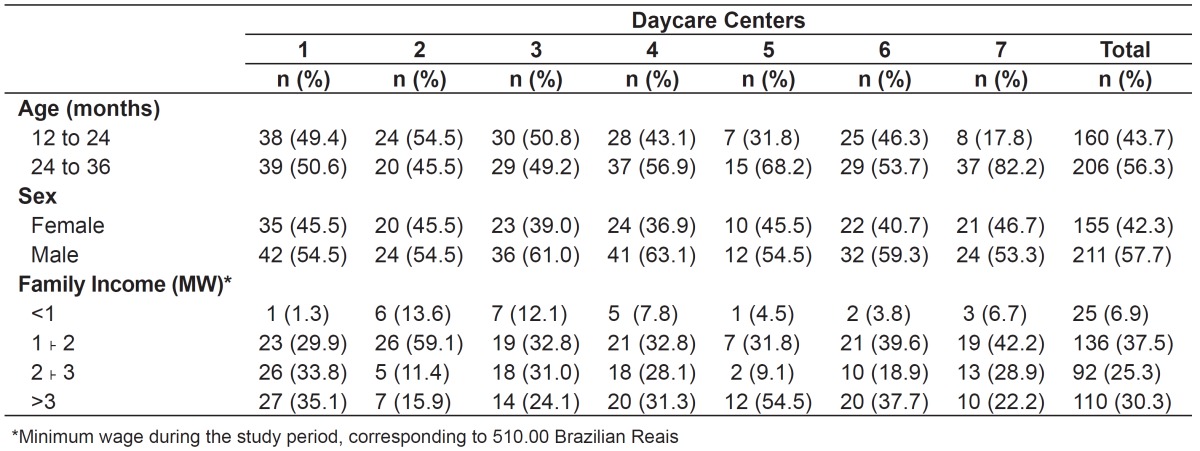
Demographic and socioeconomic characteristics of children attending public daycare centers. São Paulo, SP, 2010

The food intake based on median values and values corresponding to the first and fourth quartiles on the distribution of energy, calcium, protein, sodium, as well as the calcium/protein ratio and the density of calcium (mg/1000kcal), are expressed in Table 2, which shows equivalent values among the different nurseries, representing uniformity regarding food in public institutions.

**Table 2:**
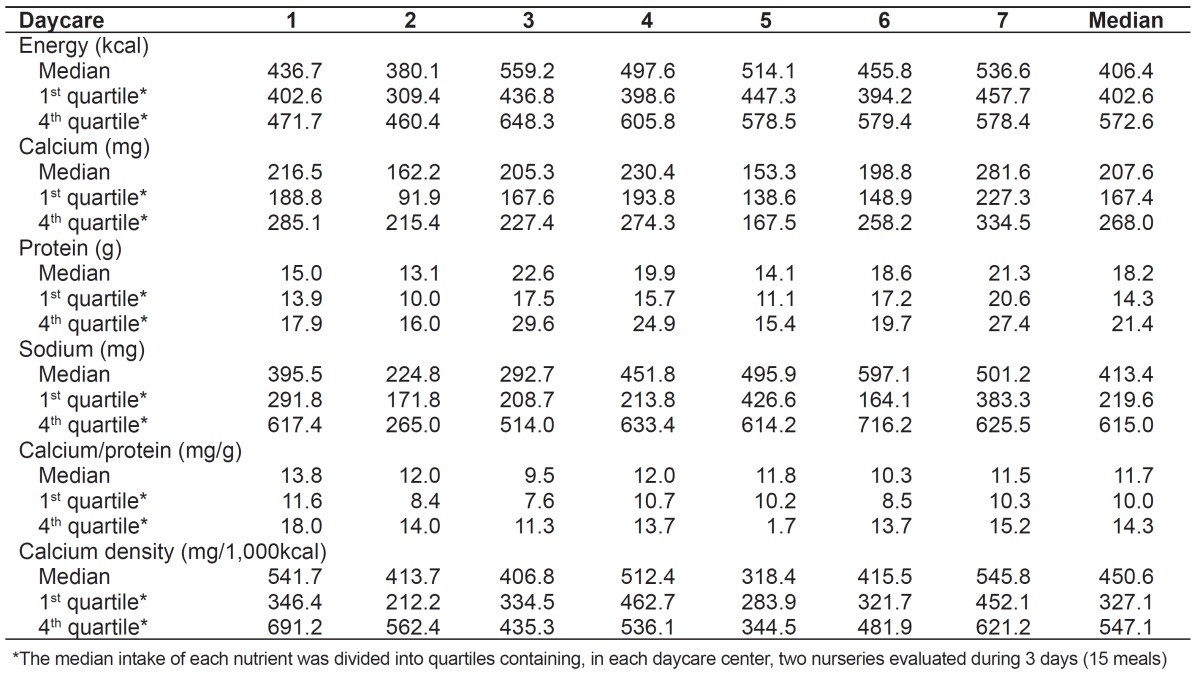
Consumption of energy, calcium, protein, sodium, calcium/protein ratio, calcium density in the diet of children attending public daycare centers. São Paulo, SP, 2010

**Figure 1:**
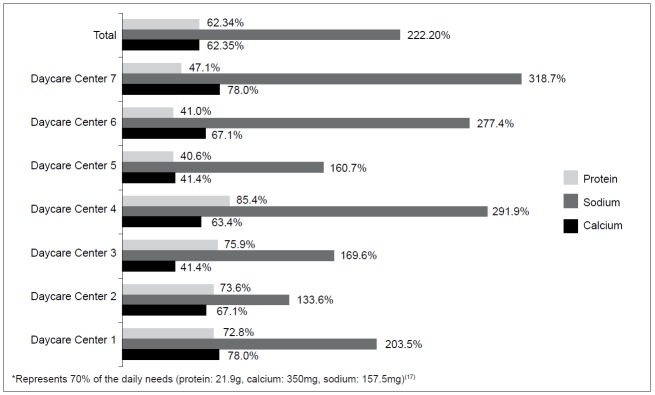
Percentage of recommended* intake of protein, calcium, and sodium in public daycare centers. São Paulo, SP, 2010

Figure 1 shows the percentage of recommended intake of protein, sodium, and calcium, demonstrating that the mean consumption of calcium and protein is below the values stipulated by PNAE; on the other hand, there was variation from 133.6 to 318.7% in the percentage of sodium adequacy.

## Discussion

The results showed that the average energy consumption was 406.4kcal, a much lower value than the expected (700kcal)^(^ 17 ^)^. Similar results were found by Spinelli et al^(^ 16 ^)^ and by Menezes and Osório^(^ 18 ^)^, who obtained, respectively, energy adequacy of 57 and 41%. Therefore, the feeding at home in the evening and in the morning is essential to complete the nutritional needs and should be guided by nutritionists, harmonizing the actions of educators in childcare centers and of parents at home.

Under this approach, the percentage of protein adequacy (62.3%) can therefore, be conditional on, the low energy consumption and, consequently, to macronutrients in general, with a contribution in total energy intake of 12.3%.

It is noteworthy that the compliance with the recommended percentage of protein contributes to growth, bone maintenance, and prevention of osteoporosis while its deficiency or excess can be deleterious. Morais and Burgos^(^ 8 ^)^ warn that adding 50g of protein in the diet increases approximately 1.6mmol in the excretion of calcium, which is considered a regulator of urinary calcium excretion, more important than the actual intake of the mineral.

In parallel, the results presented here revealed percentages of adequate intake of calcium between 41.4 and 78% in daycare centers, so the low consumption by children was generalized. Similar data was identified in a study conducted by Spinelli et al^(^ 16 ^)^ in public daycare centers in São Paulo, whose adequacy of calcium intake by children between 12 and 18 months, adjusted to 70% of needs, was of 51.7%. This percentage, lower than the recommendation, may be linked to the supply of dairy products, as, although the menu offered in these institutions provides two servings of milk (breakfast and afternoon snack), the per capita amount is insufficient, further adding the high amount of wastage, which represents an index of waste-consumption of around 50%^(^ 19 ^)^. On the same premise in Aracaju, SE, Oliveira et al^(^ 20 ^)^ found that, of 359 children from 6 to 35 months of age analyzed, 47.7% presented consumption of milk and dairy products lower than the three servings recommended for this population.

As mentioned earlier, there is strong evidence of reduced consumption of calcium in all stages of life, cultures and regions, both in developed and in developing countries. Corroborating this assertion, Rajeshwari et al^(^ 21 ^)^ followed children from 10 years to adulthood, demonstrating that calcium intake is reduced during this period, with considerable reduction from childhood (54% below the recommended level) to adulthood (77% below the recommendation). Similarly, Salamoun et al^(^ 22 ^)^, analyzing children from the Mediterranean, found that only 12% reached the recommendation for this mineral. Also, the Bogalusa Heart Study^(^ 23 ^)^ found that 69% of children did not meet the recommended intake. In the Spanish population, the amount of individuals with ingestion of calcium below the recommended levels was also found to be high, with a prevalence of 22% in preschool children^(^ 24 ^)^.

These findings are of concern due to the losses caused by the insufficient intake of calcium in childhood for the definition of the peak bone mass, considering this insufficient intake critical for the occurrence of osteoporosis in adulthood. Thus, children attending public daycare centers would be susceptible to reduced peaks of bone mass and greater risk of osteoporosis and early factures in adult life^(^ 25 ^)^. Besides the insufficient intake of calcium, the calcium-protein ratio was also inadequate: the median of this ratio in this study was of 11.8mg/g, below the <20mg/g, established as appropriate^(^ 12 ^,^ 13 ^)^. 

In this premise, Moreira et al^(^ 26 ^)^ emphasize the importance of calcium intake in relation to proteins, consigning the importance of this proportion, especially to eliminate errors in the estimates of intake. These authors found a ratio of 11.1±3.7 and 11.0±3.8g between Portuguese boys and girls, respectively. Similar data was found by Ortega et al^(^ 9 ^)^, whose proportion of nutrients was of 10.1±2.8g in a representative sample of children. 

As for sodium intake in the present study, the percentage reached from 133.6 to 318.7% of the recommended between the assessed daycare centers. This findings is disturbing, since children acquire a taste for salt according to the amount they consume daily^(^ 27 ^)^. 

The mentioned percentages, if kept frequent or rising in the diet of these children, can cause negative health impacts in the short- and long-term, once high sodium intake is known to be associated with increased prevalence of hypertension, acute myocardial infarction, renal failure, and stroke^(^ 28 ^)^. Furthermore, excess sodium is also related to the reduction of peak bone mass in adolescence and with the acceleration of bone loss input throughout life^(^ 24 ^,^ 29 ^)^.

According to Pan* et al* ^(^ 30 ^)^, sodium is among the major determinants of calcium excretion and it is estimated that a diet increased with 100mmol of sodium produces 1mmol in urinary excretion of calcium^(^ 31 ^)^. 

Besides its importance in the promotion of bone health, the early, excessive, and continued intake of a diet high in sodium contributes to increased blood pressure levels, which is considered the best predictor of blood pressure levels in adulthood. It is noteworthy that the clinical manifestation may already occur in childhood, with the prevalence of hypertension observed in Brazilian children and adolescents from 2.3 to 31%, reaching 51.7%^(^ 32 ^)^.

Despite the findings reported here, we should also mention the restrictions stipulated by PNAE on Resolution/CD/FNDE n. 38, of July, 2009^(^ 17 ^)^ in the use of sausages, sweets, pre-ready meals (or ready) or processed foods (in powder or dehydrated for reconstitution), with high amount of sodium (those with an amount equal or greater than 500mg per 100g or mL). 

It should also be emphasized that food supplementation is provided within the family context, which, according o the guidelines of the PNAE, should provide the intake of 30% of the daily nutritional needs. Although this study has not investigated household consumption, Toloni et al^(^ 33 ^)^ analyzed the introduction of processed foods in these very institutions and found that approximately two thirds of children were offered by the parents, in the home environment, instant noodles, snacks, sandwich cookies, sausages, artificial juice, soda, candy, and lollipop before 12 months of age. These data indicate the probability that the nutritional complementing, within the family context, does not contribute positively to the total adequate daily nutritional intake, emphasizing the possible availability of foods high in sodium.

Despite the inherent limitations of studies with non-probabilistic sampling procedures and biases related to different methods of dietary surveys, such as incorrect recording of answers, coding error, conversion of food into nutrients, decalibrated instruments, and inter and intrapersonal variation, it is noteworthy that the studied institutions have socioeconomic and demographic characteristics similar to the universe of public daycare centers in the municipality of São Paulo, reflecting the reality of municipal institutions. Furthermore, to minimize measurement bias, there was a training of the team, collection on non-consecutive days, calibration of equipment, as well as review and evaluation of the consistency of the instruments and the data.

Therefore, the results of this study, which reflected the inadequacy of dietary intake of calcium, protein, and sodium, are considered a first step towards awareness regarding the recommended intake for the pediatric population, especially within the context of public daycare centers. 

The action strategies in the prevention of chronic diseases related to nutrition, such as osteoporosis and hypertension, are not restricted to the medical scope and need interventions in the field of Public Health in daycare centers and schools, in the media, and other spaces where civil society lives. Thus, it is expected that the results of this study may help promote efforts that contribute to a better bone health future.

## References

[1] Bonura F (2009). Prevention, screening, and management of osteoporosis:
an overview of the current strategies. Postgrad Med.

[2] LaFleur J, McAdam-Marx C, Kirkness C, Brixner DI (2008). Clinical risk factors for fracture in postmenopausal
osteoporotic women: a review of the recent literature. Ann Pharmacother.

[3] Martínez MJ, Redondo D, Conde F, Redondo P, Alonso Franch M (2009). Gráficas longitudinales de velocidad de conducción media de
ultrasonidos en falanges.Estudio nutricional de Castilla y León.

[4] Gimeno Ballester J, Azcona San Julián  C, Sierrasesúmaga Ariznabarreta  L (2001). Bone mineral density determination by osteosonography in
healthy children and adolescents: normal values. An Esp Pediatr.

[5] Suárez Cortina L, Moreno Villares JM, Martínez V, Aranceta J, Dalmau J, Gil A (2011). Ingesta de cálcio y densidad mineral ósea en una
población de escolares españoles (estudio CADO). An Pediatr (Barc).

[6] Vue H, Reicks M (2007). Individual and environmental influences on intake of
calcium-rich food and beverages by young Hmong adolescent girls. J Nutr Educ Behav.

[7] Larson NI, Neumark-Sztainer D, Harnack L, Wall M, Story M, Eisenberg ME (2009). Calcium and dairy intake: longitudinal trends during the
transition to young adulthood and correlates of calcium intake. J Nutr Educ Behav.

[8] Morais GQ, Burgos MG (2007). Nutrients impact on bone health: new
trends. Rev Bras Ortop.

[9] Ortega RM, López-Sobaler AM, Jiménez Ortega AI, Navia Lombán B, Ruiz-Roso Calvo de Mora B, Rodríguez-Rodrígues E (2012). Food sources and average intake of calcium in a
representative sample of Spanish schoolchildren. Nutr Hosp.

[10] Longo-Silva G, Toloni MH, Goulart RM, Taddei JA (2012). Evaluation of food consumption at public day care
centers in São Paulo, Brazil. Rev Paul Pediatr.

[11] Serra-Majem L, Ribas-Barba L, Salvador G, Jover L, Raidó B, Ngo J (2007). Trends in energy and nutrient intake and risk of
inadequate intakes in Catalonia, Spain (1992-2003). Public Health Nutr.

[12] Ortega RM, López-Sobaler AM, Requejo RM, Andrés P (2010). La composición de los alimentos.Herramienta básica para la
valoración nutricional. Objetivos nutricionales marcados para la población
española. Departamento de Nutrición.

[13] Weinsier RL, Krumdieck CL (2000). Dairy foods and bone health: examination of the
evidence. Am J Clin Nutr.

[14] Konstantyner T, Taddei JA, Oliveira MN, Palma D, Colugnati FA (2009). Isolated and combined risks for anemia in children
attending the nurseries of daycare centers. J Pediatr (Rio J).

[15] Alves G, Colauto EV, Fernandes JK, Zabine L, Nienow RC (2008). Anthropometric and food intake assessment of
preschoolers in day-care centers in Umuarama, Paraná. Arq Ciências Saúde Unipar.

[16] Spinelli MG, Goulart RM, Santos AL, Gumiero LD, Farhud CC, Freitas EB (2003). Six to eighteen-month-old children's food intake in
day-care centers. Rev Nutr.

[17] Brasil - Ministério da Educação. Fundo Nacional de Desenvolvimento da
Educação. Conselho Deliberativo (2009). Resolução/CD/FNDE nº 38, de 16 de julho de 2009. Dispõe sobre o
atendimento da alimentação escolar aos alunos da educação básica no Programa
Nacional de Alimentação Escolar - PNAE.

[18] Menezes RC, Osório MM (2007). Energy and protein intake and nutritional status of
children under five years of age in Pernambuco state, Brazil. Rev Nutr.

[19] Longo-Silva G, Toloni MH, Rocha AM, Rodrigues S, Taddei JA (2012). Evaluation of the menu, food consumption and food waste
in outsourced public daycare centers in Brazil. Nutrícias.

[20] Oliveira S, Filha E, Araújo JS, Barbosa JS, Gaujac DP, Santos CF, Silva DG (2012). Consumption of food groups among children attending the
public health system of Aracaju, Northeast Brazil, in Sergipe. Rev Paul Pediatr.

[21] Rajeshwari R, Nicklas TA, Yang SJ, Berenson GS (2004). Longitudinal changes in intake and food sources of
calcium from childhood to young adulthood: the Bogalusa Heart
Study. J Am Coll Nutr.

[22] Salamoun MM, Kizirian AS, Tannous RI, Nabulsi MM, Choucair MK, Deeb ME (2005). Low calcium and vitamin D intake in healthy children and
adolescents and their correlates. Eur J Clin Nutr.

[23] Nicklas TA (2003). Calcium intake trends and health consequences from
childhood through adulthood. JACN.

[24] Ortega RM, Requejo AM, Navia B, Quintas ME, Andrés P, López-Sobaler M (2000). The consumption of milk products in a group of
pre-school children: influence on serum lipid profile. Nutr Res.

[25] Tucker KL (2003). Does milk intake in childhood protect against later
osteoporosis. Am J Clin Nutr.

[26] Moreira P, Padez C, Mourão I, Rosado V (2005). Dietary calcium and body mass index in Portuguese
children. Eur J Clin Nutr.

[27] Ramos M, Stein LM (2000). Desenvolvimento do comportamento alimentar
infantil. J Pediatr (Rio J).

[28] Costa FP, Machado SH (2010). Does the consumption of salt and food rich in sodium
influence in the blood pressure of the infants. Cien Saude Colet.

[29] Cappuccio FP, Kalaitzidis R, Duneclift S, Eastwood JB (2000). Unravelling the links between calcium excretion, salt
intake, hypertension, kidney stones and bone metabolismo. J Nephrol.

[30] Pan W, Borovac J, Spicer Z, Hoenderop JG, Bindels RJ, Shull GE (2012). The epithelial sodium/proton exchanger, NHE3, is
necessary for renal and intestinal calcium (re)absorption. Am J Physiol Renal Physiol.

[31] Cirillo M, Ciacci C, Laurénzi M, Mellone M, Mazzacca G, De Danto NG (1997). Salt intake, urinary sodium, and
hypercalciuria. Miner Eletrolyte Metab.

[32] Bezerra ML, Soares PF, Leite ES, Lucena RC (2013). Hypertension in children and adolescents: a systematic
review about prevalence and risk factors. Rev Enferm UFPE.

[33] Toloni MH, Longo-Silva G, Goulart RM, Taddei JA (2011). Introduction of processed and traditional foods to the
diets of children attending public daycare centers in São Paulo,
Brazil. Rev Nutr.

