# Supplementary material for: Intake of protein, calcium and sodium in public child day care centers
Source: Rev Paul Pediatr. 2014 Jun;32(2):193–9. doi: 10.1590/0103-0582201432214613 (PMC4183027; doi:10.1590/0103-0582201432214613)
Supplement: Translation

# Translation: Ingestão de proteína, cálcio e sódio em creches públicas

**Authors:** Giovana Longo-Silva, Maysa Helena de A. Toloni, Risia Cristina E. de Menezes, Tatiane Leocádio Temteo, Maria Alice A. Oliveira, Leiko Asakura, Emília Chagas Costa, José Augusto de A. C. Taddei

**Affiliations:** 1 Faculdade de Nutrição da Universidade Federal de Alagoas (UFAL), Maceió, AL, Brasil; 2 Escola Paulista de Medicina da Unifesp, São Paulo, SP, Brasil; 3 Universidade Federal de Pernambuco (UFPE), Recife, PE, Brasil

**Keywords:** criança, osteoporose, cálcio na dieta, sódio na dieta, creches, saúde escolar

## Abstract

**OBJETIVO:**

Avaliar a ingestão de cálcio, proteína e sódio e compará-la com a recomendada, em crianças que frequentam creches públicas.

**MÉTODOS:**

Estudo transversal descritivo realizado em berçários de sete creches públicas do município de São Paulo, que atendiam 366 crianças entre 12 e 36 meses, com coleta entre setembro a dezembro de 2010. Avaliou-se cada creche durante três dias não consecutivos, totalizando 42 dias e 210 refeições. O consumo alimentar foi avaliado por método de pesagem direta de alimentos. Para o cálculo nutricional, utilizou-se o *software DietWin Profissional* 2.0

**(r):**

e a adequação foi calculada segundo as recomendações do Programa Nacional de Alimentação Escolar (PNAE) para energia, proteína, cálcio e sódio. A relação cálcio/proteína e a densidade de cálcio (mg/1.000kcal) também foram computadas.

**RESULTADOS:**

Constatou-se que o consumo de energia (406,4kcal), de proteína (18,2g) e de cálcio (207,6mg) não atingiu os valores recomendados em todas as creches estudadas, sendo que a ingestão de sódio ultrapassou em até três vezes a recomendação. A relação cálcio/proteína de 11,7mg/g foi inferior à adequação (20mg/g).

**CONCLUSÕES:**

Há inadequação do consumo alimentar de cálcio, proteína e sódio, em crianças inseridas no contexto de creches públicas.

## Introdução

No último século, a expectativa de vida tem aumentado consideravelmente em países tanto desenvolvidos quanto em desenvolvimento, o que tem favorecido um incremento notável na incidência de enfermidades relacionadas à idade, entre elas, a osteoporose^(^ ^1^ ^)^. O risco de sofrer fraturas osteoporóticas ao longo da vida chega a 40% entre as mulheres e 13% entre os homens^(^ ^2^ ^)^. A manutenção da saúde óssea é, portanto, um dos grandes desafios da Medicina moderna.

Uma vez estabelecida a enfermidade, é muito difícil revertê-la. Apesar de toda estratégia assistencial, as ações de prevenção devem ser priorizadas, enfatizando a importância da mineralização óssea nas primeiras etapas da vida, já que a aquisição de massa óssea ocorre principalmente na infância e a realização desse pico depende de múltiplos fatores, sendo a nutrição um dos mais importantes^(^ ^3^ ^,^ ^4^ ^)^.

Embora muitos nutrientes estejam envolvidos na manutenção da saúde óssea, o cálcio é especialmente importante durante os períodos de crescimento acelerado, como a infância e a adolescência^(^ ^5^ ^-^ ^7^ ^)^, sendo a sua contribuição fundamental na prevenção de diversas enfermidades em fases posteriores da vida, como obesidade, hipertensão, resistência à insulina, cálculos renais e câncer de cólon^(^ ^6^ ^,^ ^7^ ^)^. Por outro lado, a ingestão reduzida faz com que o mineral já utilizado no osso seja remanejado para outras funções fisiológicas vitais, como a manutenção da calcemia^(^ ^8^ ^)^.

Apesar da importância para a Saúde Pública de se alcançar uma ingestão adequada de cálcio, inúmeras pesquisas indicam que o seu aporte e o consumo de produtos lácteos, principais fontes desse mineral^(^ ^6^ ^)^, são frequentemente inferiores aos recomendados para crianças, sobretudo no contexto das creches públicas^(^ ^9^ ^-^ ^11^ ^)^. No entanto, para avaliar o consumo de cálcio, deve-se considerar a influência de outros nutrientes que atuam sobre sua absorção e utilização. Nesse sentido, ao seu aporte suficiente se soma o consumo adequado de sódio e a relação cálcio-proteína, sendo estabelecida como conveniente a proporção <20mg/g^(^ ^12^ ^,^ ^13^ ^)^.

Assim, o objetivo deste estudo foi avaliar a ingestão de proteína, cálcio e sódio e compará-la com a recomendada, considerando-se esse passo importante para promover futuras melhorias nutricionais na alimentação escolar.

## Método

Este estudo é parte integrante do "Projeto CrechEficiente - Impacto do treinamento de educadores de creches públicas/filantrópicas nas práticas higiênico-dietéticas e na saúde/nutrição dos lactentes, cujos objetivos foram capacitar, aperfeiçoar e atualizar os educadores de creches quanto aos cuidados de saúde e de nutrição oferecidos aos lactentes, bem como avaliar a aquisição de conhecimentos pelos educadores relacionados às atividades desenvolvidas. O processo de seleção das creches e os critérios adotados estão descritos em outra publicação^(^ ^14^ ^)^. Das oito creches selecionadas, uma foi excluída por não apresentar interesse em participar da pesquisa no período de coleta dos dados aqui apresentados.

Este trabalho é do tipo transversal, descritivo, desenvolvido nos 14 berçários das sete creches selecionadas. A coleta de dados ocorreu de setembro a dezembro de 2010 e foi realizada por quatro nutricionistas pós-graduandas, pesquisadoras da Universidade Federal de São Paulo (Unifesp). 

Para avaliar o consumo alimentar, utilizou-se a pesagem direta dos alimentos, para garantir a precisão e a acurácia dos resultados, considerando-se a média de ingestão do grupo. Tal metodologia tem sido utilizada repetidamente em creches brasileiras, constituindo-se, portanto, em instrumento de eleição para essa finalidade^(^ ^10^ ^,^ ^15^ ^,^ ^16^ ^)^.

Todas as instituições funcionavam em período integral, de segunda a sexta-feira, e avaliaram-se todas as refeições servidas (desjejum, lanche da manhã ou hidratação, almoço, lanche da tarde e jantar), durante três dias não consecutivos, totalizando 42 dias de análise e 210 refeições.

A pesagem de alimentos sólidos foi realizada com balança eletrônica digital portátil Plenna^(r)^, com capacidade de 5kg. Os alimentos líquidos foram medidos com o auxílio de recipiente graduado (copo plástico) a cada 50mL e com capacidade para 1.000mL.

Pesaram-se três porções de cada alimento e/ou preparação, selecionados aleatoriamente, para se obter a quantidade média servida. Coletaram-se os alimentos não consumidos em sacos plásticos, considerando-se como resto todo alimento e/ou preparação que a criança deixava no copo ou prato. Acrescenta-se que, para se obter o peso da refeição rejeitada, coletaram-se os pesos individualmente para cada alimento e/ou preparação, servidos no café da manhã, lanche da manhã, lanche da tarde e, ainda, no jantar, quando composto unicamente por sopa. Porém, no almoço e no jantar, refeições nas quais os alimentos são servidos no prato e misturados, a análise considerou o resto proporcional para cada alimento e/ou preparação.

Obtendo-se a média dos alimentos e/ou preparações servidas, multiplicou-se o valor pelo número de crianças de cada grupo. As repetições foram somadas ao valor total, obtendo-se, dessa forma, o peso total de cada alimento e/ou preparação. Desse valor, subtraiu-se o resto de cada alimento servido e, por fim, dividiu-se pelo número de crianças de cada grupo, obtendo-se, assim, a quantidade *per capita* de cada alimento e/ou preparação. Para o cálculo do valor nutritivo das refeições, utilizou-se o *software DietWin Profissional* 2.0^(r)^.

Calculou-se o percentual de recomendação para cálcio, proteína e sódio, considerando-se os valores de referência estipulados pelo Programa Nacional de Alimentação Escolar (PNAE) - 70% das necessidades nutricionais diárias^(^ ^17^ ^)^. 

Devido à ausência de receituário padrão para o preparo de sopas, leites e sucos, a equipe acompanhou a preparação, pesando previamente todos os ingredientes utilizados e o rendimento final. A informação nutricional de cada preparação também foi calculada no *software DietWin Profissional* 2.0^(r)^ e, posteriormente, inserida como uma nova preparação na lista de alimentos do programa. 

Para determinar o perfil socioeconômico das crianças frequentadoras das instituições analisadas, aplicou-se aos pais um questionário para identificar a renda familiar de cada criança, obtida por meio de informações sobre salários e outras fontes de renda de todos os membros da unidade familiar. A somatória obtida foi expressa em reais e convertida em unidades de salário mínimo (SM) vigente no período do estudo. Descreveram-se as frequências simples e percentuais da distribuição da idade, do sexo e da classe econômica das crianças. 

Todos os dados obtidos foram duplamente digitados, validados e analisados no programa estatístico Epi-Info 2000, versão 3.4.3. O projeto foi aprovado pelo Comitê de Ética em Pesquisa da Unifesp.

## Resultados

A caracterização socioeconômica das crianças matriculadas nas creches avaliadas está contemplada na Tabela 1, notando-se que a distribuição do sexo das crianças frequentadoras das sete creches estudadas foi homogênea, havendo 366 crianças com idades entre 12 e 36 meses. As condições econômicas das famílias atendidas nesse serviço apontam que 62,8% têm renda familiar variando entre um e três salários mínimos. 

**Tabela 1:** Características demográficas e socioeconômicas de crianças matriculadas em creches públicas. São Paulo, SP, 2010

O consumo alimentar baseado nos valores da mediana e valores correspondentes ao primeiro e quarto quartil da distribuição de energia, cálcio, proteína, sódio, bem como a relação cálcio/proteína e a densidade de cálcio (mg/1.000kcal), estão expressos na Tabela 2, na qual se observa homogeneidade nos valores entre as diferentes creches, representando uniformidade quanto à alimentação em instituições públicas.

**Tabela 2:** Consumo de energia, cálcio, proteína, sódio, relação cálcio/proteína e densidade de cálcio na dieta de crianças frequentadoras de creches públicas. São Paulo, SP, 2010

Na Figura 1, verifica-se o percentual de recomendação de proteína, sódio e cálcio, constatando-se que o consumo médio de cálcio e de proteína encontra-se abaixo dos valores estipulados pelo PNAE; por outro lado, houve variação de 133,6 a 318,7% no percentual de adequação de sódio.

**Figura 1:** Percentual de recomendação de proteína, cálcio e sódio em creches públicas. São Paulo, SP, 2010

## Discussão

Os resultados mostraram que a mediana do consumo de energia foi de 406,4kcal, valor muito inferior ao planejado (700kcal)^(^ ^17^ ^)^. Resultados semelhantes foram encontrados por Spinelli *et al**^(^* ^16^ ^)^ e por Menezes e Osório^(^ ^18^ ^)^, que obtiveram, respectivamente, adequação de energia de 57 e 41%. Assim, a alimentação em casa, durante a noite e pela manhã, é indispensável para se completarem as necessidades nutricionais e deve ser orientada por nutricionistas, harmonizando a atuação de educadores nas creches e dos pais nas residências.

Sob esse enfoque, o percentual de adequação proteica (62,3%) pode se condicionar, portanto, ao baixo consumo energético e, consequentemente, aos macronutrientes de forma generalizada, sendo sua contribuição no aporte total energético de 12,3%.

Salienta-se que o atendimento à recomendação percentual de proteína contribui para o crescimento, a manutenção óssea e a prevenção da osteoporose e sua deficiência ou excesso podem ser deletérios. Morais e Burgos^(^ ^8^ ^)^ alertam que o incremento na dieta de 50g de proteína eleva aproximadamente 1,6mmol na excreção de cálcio, sendo considerada um regulador da excreção urinária de cálcio, mais importante que a própria ingestão do mineral.

Paralelamente, os resultados aqui apresentados revelaram percentuais de adequação de cálcio entre 41,4 e 78% nas creches, sendo, portanto generalizado o seu baixo consumo pelas crianças. Identificou-se dado congênere em estudo conduzido por Spinelli *et al**^(^* ^16^ ^)^ em creches públicas de São Paulo, cuja adequação do consumo de cálcio por crianças entre 12 e 18 meses, ajustada para 70% das necessidades, foi de 51,7%. Esse percentual, inferior à recomendação, pode estar condicionado à oferta de lácteos, pois, embora o cardápio oferecido nessas instituições preveja a oferta de duas porções de leite (desjejum e lanche da tarde), a quantidade *per capita* é insuficiente, acrescentando-se ainda o elevado desperdício, o que representa um índice de resto-ingestão em torno de 50%^(^ ^19^ ^)^. Na mesma premissa, em Aracaju, SE, Oliveira *et al**^(^* ^20^ ^)^ constataram que, das 359 crianças de seis a 35 meses de idade estudadas, 47,7% apresentavam consumo de leite e derivados inferior às três porções recomendadas a essa população.

Como mencionado anteriormente, observa-se forte tendência de redução do consumo de cálcio em todas as fases do curso da vida, culturas e regiões, tanto em países desenvolvidos como em desenvolvimento. Corroborando essa asserção, Rajeshwari *et al**^(^* ^21^ ^)^ acompanharam crianças dos dez anos até a vida adulta, demonstrando que o consumo de cálcio é diminuído durante esse período, havendo redução considerável da infância (54% abaixo da recomendação) à idade adulta (77% abaixo da recomendação). De forma semelhante, Salamoun *et al**^(^* ^22^ ^)^, analisando crianças do Mediterrâneo, constataram que apenas 12% atingiam a recomendação para esse mineral. Ainda, o *Bogalusa Heart Study**^(^* ^23^ ^)^ identificou que 69% das crianças não atendiam à ingestão recomendada. Na população espanhola, também se constatou ser elevado o percentual de indivíduos com ingestão de cálcio abaixo da recomendação, sendo a prevalência de 22% nos pré-escolares^(^ ^24^ ^)^.

Tais achados tornam-se preocupantes em decorrência dos prejuízos da ingestão insuficiente de cálcio na infância e na adolescência para a definição do pico de massa óssea, considerando-se esse aporte insuficiente fator crítico para a ocorrência de osteoporose na vida adulta. Assim, as crianças frequentadoras de creches públicas estariam suscetíveis a picos reduzidos de massa óssea e a maior risco de osteoporose e de fraturas precoces na vida adulta^(^ ^25^ ^)^. Acrescenta-se que, ao aporte insuficiente de cálcio, se soma a relação cálcio-proteína que não foi adequada: a mediana dessa relação no presente estudo foi de 11,8mg/g e, portanto, <20mg/g, que é o estabelecido como apropriado^(^ ^12^ ^,^ ^13^ ^)^. 

Nessa premissa, Moreira *et al**^(^* ^26^ ^)^ enfatizam a importância da ingestão de cálcio em relação às proteínas, consignando a importância dessa proporção, sobretudo para eliminar erros nas estimativas de ingestão. Esses autores encontraram em crianças portuguesas uma relação de 11,1±3,7 e 11,0±3,8g entre meninos e meninas, respectivamente. Dado semelhante foi constatado por Ortega *et al**^(^* ^9^ ^)^, cuja proporção dos nutrientes foi de 10,1±2,8g em uma amostra representativa de crianças. 

Quanto ao consumo de sódio, no presente estudo, os percentuais atingiram de 133,6 a 318,7% do recomendado entre as creches avaliadas. Esse dado é inquietante, uma vez que as crianças adquirem o gosto por sal conforme a quantidade que ingerem diariamente^(^ ^27^ ^)^. 

As porcentagens mencionadas, caso mantenham-se frequentes ou ascendentes na dieta dessas crianças, podem causar impactos negativos à saúde em curto e longo prazo, visto que o consumo elevado de sódio está sabidamente associado ao aumento das prevalências de hipertensão arterial, infarto agudo do miocárdio, insuficiência renal e acidente vascular encefálico^(^ ^28^ ^)^. Além disso, o excesso de sódio relaciona-se ainda com a redução do pico da massa óssea na adolescência e com a aceleração da perda de massa óssea ao longo da vida^(^ ^24^ ^,^ ^29^ ^)^.

De acordo com Pan* et al**^(^* ^30^ ^)^, o sódio está entre os maiores determinantes da excreção de cálcio, estimando-se que uma dieta com aumento de 100mmol de sódio produz aumento de 1mmol na excreção urinária do cálcio^(^ ^31^ ^)^. 

Além da sua importância para se promover a saúde óssea, o consumo precoce, excessivo e continuado de uma dieta com elevado teor de sódio contribui para a elevação dos níveis de pressão arterial, sendo este considerado o maior preditor dos níveis pressóricos na fase adulta. Ressalta-se que a manifestação clínica pode ocorrer ainda na infância, sendo a prevalência de hipertensão observada em crianças e adolescentes brasileiros de 2,3 a 31%, chegando a atingir 51,7%^(^ ^32^ ^)^.

Apesar da constatação aqui relatada, devem-se ressaltar as restrições estipuladas pelo PNAE na resolução/CD/FNDE nº 38, de 16 de julho de 2009^(^ ^17^ ^)^, na utilização de alimentos enlatados, embutidos, doces, preparações semiprontas (ou prontas) ou alimentos processados (em pó ou desidratados para reconstituição), com quantidade elevada de sódio (aqueles que possuem em sua composição quantidade igual ou superior a 500mg por 100g ou mL). 

Cumpre ainda ressaltar que é prevista a complementação alimentar no contexto familiar, onde, segundo as diretrizes do PNAE, deve haver o aporte de 30% das necessidades nutricionais diárias. Embora o presente estudo não tenha investigado o consumo domiciliar, Toloni *et al**^(^* ^33^ ^)^ analisaram a introdução de alimentos industrializados nessas mesmas instituições e constataram que, para aproximadamente dois terços das crianças, foram oferecidos, pelos pais, no ambiente domiciliar, macarrão instantâneo, salgadinhos, bolacha recheada, embutidos, suco artificial, refrigerante, bala e pirulito antes dos 12 meses de idade. Tais dados indicam a probabilidade de a complementação, no contexto familiar, não contribuir positivamente para a adequada ingestão nutricional total diária, enfatizando a possível disponibilidade de alimentos ricos em sódio.

Apesar das limitações inerentes aos estudos com procedimento amostral não probabilístico e vieses relacionados aos distintos métodos de inquéritos alimentares, tal como registro incorreto de respostas, erro de codificação, conversão de alimentos em nutrientes, instrumentos descalibrados e variação inter e intrapessoal, ressalta-se que as instituições estudadas apresentam características socioeconômicas e demográficas similares ao universo das creches públicas inseridas no município de São Paulo, refletindo a realidade das instituições municipais. Ademais, para minimizar os vieses de aferição, houve treinamento da equipe, coleta em dias não consecutivos, calibração dos equipamentos, bem como revisão e avaliação da consistência dos instrumentos e dos dados.

Assim, os resultados deste estudo, que refletiram a inadequação de consumo alimentar de cálcio, proteína e sódio, são considerados um primeiro passo de conscientização e aproximação da ingestão recomendada para a população infantil, sobretudo inserida no contexto de creches públicas. 

As estratégias de atuação na prevenção de doenças crônicas relacionadas à alimentação, como a osteoporose e a hipertensão, não se restringem ao escopo médico e necessitam de intervenções no âmbito da Saúde Pública, em creches e escolas, meios de comunicação e outros locais onde vive a sociedade civil. Assim, espera-se que os resultados do presente estudo possam auxiliar na promoção de esforços que contribuam para uma melhor saúde óssea futura.
